# Collagen properties of Indonesian local sheepskin isolated using acid and enzymatic methods

**DOI:** 10.5455/javar.2024.k823

**Published:** 2024-09-29

**Authors:** Dita Prameswari Trenggono Putri, Vernanda Widya Pangestika, Hanifan Ilyas, Mohammad Zainal Abidin, Nanung Agus Fitriyanto, Yuny Erwanto

**Affiliations:** 1 Department of Animal Products Technology, Faculty of Animal Science, Gadjah Mada University, Yogyakarta, Indonesia; 2 Institute for Halal Industry and System, Gadjah Mada University, Yogyakarta, Indonesia

**Keywords:** Enzymatic method, Acid method, Collagen, Characteristics, Garut Sheep

## Abstract

**Objectives:**

This study aimed to investigate the physical properties of Indonesian local sheep skin collagen extracted by acid and enzymatic methods.

**Materials and Methods:**

Collagen was isolated from Pure Breed Garut Sheep (*Ovis aries sp*.) skin, 1.5 years old. The skins were obtained from a local slaughterhouse in Cirebon, Indonesia. The solvents used were CH_3_COOH and three different enzymes: neutrase, alcalase, and bromelain.

**Results:**

The highest yields of extracted collagen were bromelain-soluble collagen (BSC), which reached 37.07%. The range of Ph values for all samples started from 4.01 to 4.76. The viscosity values (cP) of acid-soluble collagen (ASC), neutrase-soluble collagen (NSC), alcalase-soluble collagen (LSC), and BSC were 3.42, 3.90, 3.45, and 3.12, respectively. Regarding SDS-PAGE analyses, Garut sheepskin collagen is categorized as collagen type I, which has a molecular weight of about 140.99 to 148.74 kDa for α1 and around 110 to 111.86 kDa for α2. The results of FTIR and DSC analyses for all samples show the same motif with commercial collagen motifs based on the literature.

**Conclusion:**

Garut sheep skin has the potential to be an alternative raw material source for producing collagen. Collagen extracted using a combination of CH3COOH and bromelain enzyme showed the most desirable results in almost every characteristic.

## Introduction

Garut sheep is one of Indonesia’s local sheep breeds spread in West Java Province and has been cultivated for generations by the local community [1]. Garut sheep result from a cross between Merino sheep from Australia and Kaapstad sheep from South Africa with local sheep [2]. The skin weight of sheep is about 7 to 13% of the body weight [3]. Not all Garut sheep skin, a by-product of livestock slaughter, has been utilized properly, so efforts must be made to utilize it. Matinong et al. [4] stated that animal skin contains 60% collagen in dry weight. Thus, it has the potential to be used as a raw material for collagen protein sources.

Collagen is the most abundant triple helix structural protein in all animals. Collagen can be found in skin, tendons, bones, cartilage, ligaments, and other organ tissues. So far, ~28 different types of collagen have been identified [5]. Along with the development of technology and lifestyle, collagen products are widely utilized as raw materials for the food, pharmaceutical, biomedical, and cosmetic industries [6]. In some countries, religious and cultural issues affect consumers’ preferences in their consumption. In Indonesia, most people are Muslim and concerned about halal products. On the other hand, imported collagen in Indonesia is very high. However, many products are not confirmed as halal products, so an alternative raw material source to produce collagen is needed. Garut sheepskin, a by-product of Indonesian local livestock slaughtering, can be one of the alternative raw material sources to produce good-quality collagen.

Collagen hydrolysis using enzymes has the advantage that the reaction conditions are milder than acid or base hydrolysis, and the products are relatively controllable and predictable because digestive enzymes usually cleave certain peptide bonds specifically. However, pretreatment before hydrolysis is still needed and is usually exposed to acid to help the enzyme work effectively [7]. In the previous study, many proteases from different sources were used to break down large molecular proteins into smaller molecules, such as neutrase [8], alcalase [9], bromelin, and pepsin [10]. The study about collagen isolation with bromelin is still limited. Furthermore, isolation with neutase and alcalase enzymes is never done. Enzymes from plants and bacteria are also could be an alternative solution for Halal enzyme sources.

Thus, the objective of the present study was to extract and characterize the collagen, both acid-soluble collagen (ASC) and enzyme-soluble collagen, from the skin of Garut Sheep, using a different source of protease including neutrase (NSC), alcalase (LSC) bromelain (BSC).

## Materials and Methods

### Ethical approval

This study did not involve any analysis that required ethical approval.

### Materials

Eighteen-month-old purebred male Garut sheep skin was obtained from the local farmer and preserved by ice during transportation. Neutrase (≥0.8 U/gm), alcalase (2.4 U/gm), and bromelain (3000 U/gm) were purchased from Sigma-Aldrich. All reagents used in this study were analytical grade from Sigma-Aldrich.

### Sample preparation

The sample was prepared according to Hakim et al. [11] with some modifications. The hair of the sheepskin was removed, followed by a fleshing process to remove the fat and leftover meat. The skins were cut into small pieces and weighed 50 gm for every sample pouch. Skin samples were stored in the freezer at −18°C until the sample was ready to be used for extraction.

### Isolation of Garut sheepskin collagen

The extraction method of the samples was performed according to Wahyuningsih et al. [12] with some modifications. Briefly, 50 gm of skin was soaked in a 0.1 M NaOH solution at a ratio of 1:10 (w/v) for 24 h at 4°C to remove non-collagenous proteins, and then the solution was discarded. The sheepskin was soaked into the mixture solution of 0.5% (w/w) protease enzyme (neutrase, alcalase, or bromelain) and 0.5 M CH_3_COOH for 24 h at 4°C. After that, the extracted sheepskin was squeezed, and the solution was collected.

### Collagen precipitation and dialysis

The precipitation process was conducted according to Hakim et al. [11] method with some slight modifications. The extract solution was precipitated by 2.6 M NaCl for 12 h, followed by centrifugation at 10.000 rpm for 30 min at 4°C. After centrifugation, the precipitate was redissolved in 0.5 M CH_3_COOH at 1:5 (w/v). The solution was then dialyzed by 0.1 M CH_3_COOH, followed by distilled water for 24 h (exchange solution every 3 h). The solution was lyophilized to obtain dry collagen.

### Determination of collagen yield

The yield of collagen was measured using the Akram and Zhang [13] method, which can be calculated by dividing the results by the initial weight of the sample (50 gm). The percentage of yield can be calculated with the following formula:

Collagen yields (%) = (wet weight of collagen / initial weight) × 100%.

### pH

The pH value was analyzed using the Devita method [10]. It was determined by checking the wet collagen sample with a pH meter. The pH meter tool was activated and then left to stabilize. The pH meter electrode was then briefly immersed in the sample until the pH value obtained was steady and constant.

### FTIR analysis

The FTIR analysis was performed according to Li et al. [6] 500 cm^-1^. The functional groups of samples were determined based on the detected wavenumber absorption peaks with the absorption region for protein functional groups.

### Molecular weight analysis (SDS PAGE)

The SDS PAGE analysis was performed according to Kuwahara [14] with some modifications. A 25 mg collagen sample was dissolved in 1 ml CH3COOH (0.1M). A vortex was then used to homogenize the solution. An amount of 10 μl collagen solution was combined with 4 μl loading SDS buffer before being heated in a 90°C water bath for 5 min and cooled. Protein marker and sample were added to the SDS gel. Then, for 150 min, samples were electrophoresed at 150 V and 40 mA. Finally, the gel was stained with 0.25% Coomassie blue for 15 min and then rinsed with water for a night.

### DSC analysis

The thermal stability of the sample was evaluated according to the method of Liu et al. [15]. Thermal analysis was performed using DSC. Around 5 to 10 mg samples were placed in an aluminum container and sealed. The samples were then examined at temperatures ranging from 20°C to 300°C at 10°C per min. The graph shows the characteristic isothermal peaks of collagen.

### Viscosity analysis

Viscosity measurement was performed according to [16]. Briefly, 0.3 gm of samples were diluted in a 0.1 M CH_3_COOH solution. After that, a viscometer evaluated the solution using 100 rpm speed and spindle no. 1.

### Experimental design and data analysis

The experimental design was completely randomized with one-way ANOVA analysis. Data on yield, pH, and viscosity were performed in triplicate and analyzed by analysis of variance followed by Duncan’s New Multiple Range Test if the results were significant. Data on SDS PAGE, DSC, and FTIR analysis were analyzed using descriptive analysis. Statistical analysis calculations were performed with the help of SPSS software.

## Results 

### The molecular weight of collagen

**Figure 1.**
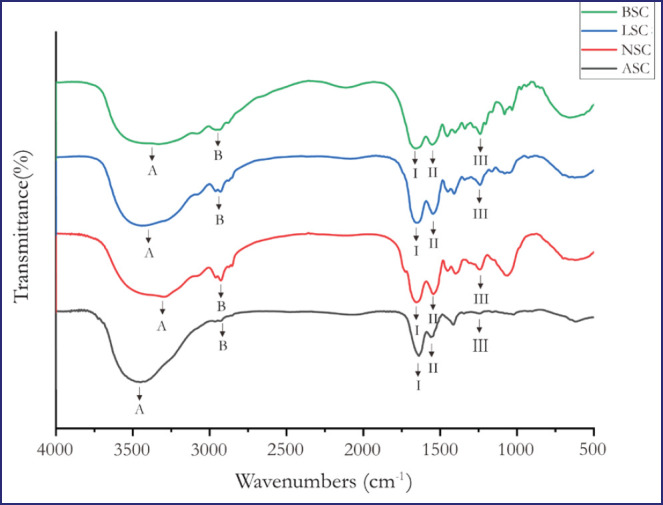
SDS-PAGE results of collagen extracted with various enzymes and CH_3_COOH. A. (1) marker, (2) neutrase, (3) alcalase, (4) bromelain, (5) CH_3_COOH, (R) commercial collagen Type I (SIGMA).

The molecular weight of Garut sheepskin collagen extracted using the acid and enzymatic methods is shown in Figure 1. All the samples have the same bands characteristic of type I collagen, which show α1 and α2 chains. Table 1 shows the molecular weights of the α1 chains of NSC, LSC, BSC, and ASC were 148.74, 145.01, 142.69, and 140.99 kDa, respectively. Furthermore, the molecular weights of the α2 chains of NSC, LSC, BSC, and ASC were 111.86, 110.99, 110, and 110.23 kDa, respectively. There was a slightly different motif among all the samples. Neutrase-soluble collagen (NSC) has the highest molecular weight, either α1 and α2 chains. On the other hand, ASC has the lowest molecular weight for the α1 chain and BSC for α2 chain.

**Table 1.** Molecular weight profile using SDS-PAGE of Indonesian local sheepskin collagen.

Band	NSC	LSC	BSC	ASC	Commercial collagen type I (SIGMA) [22]
α1 (kDa)	148,74	145,01	142,69	140,99	120
α2 (kDa)	111,86	110,99	110	110,23	116

**Figure 2.**
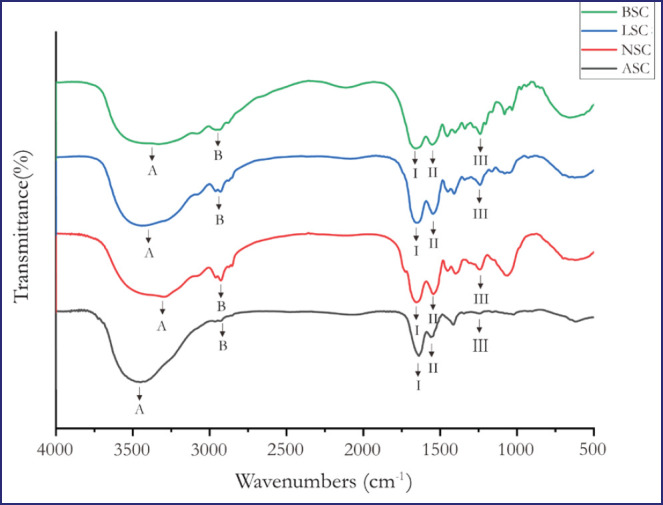
FTIR spectra of collagen extracted with acid and various enzymatic methods.

### FTIR analysis

Figure 2 and Table 2 display the peak positions of collagen’s infrared spectra. It can be seen that the motif of the functional group in extracted Garut sheep collagen using acid and enzymatic methods has five amino acid bands, including amino acids A, B, I, II, and III, which were characterized as collagen structures. The spectra of all collagen samples showed peaks in regions 3320 to 3458 for amide A, 2924 to 2960 for amide B, 1643 to 1661 for amide I, 1535 to 1550 for amide II, and 1240 to 1245 for amide III.

### DSC analysis

**Figure 3.**
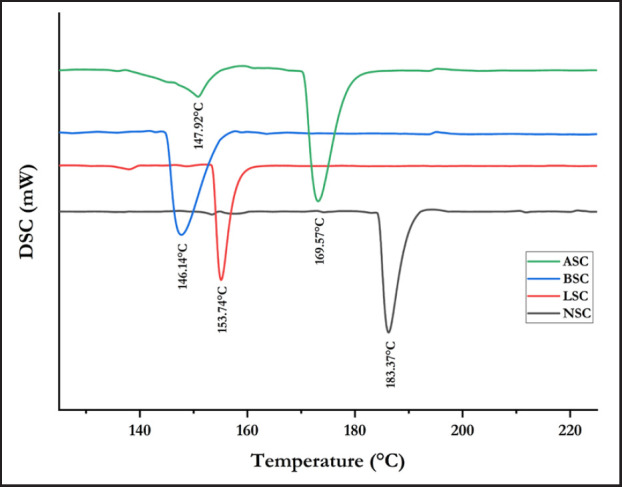
DSC spectra of collagen samples extracted with acid and various enzymatic methods.

The results of the DSC analysis of the samples can be seen in Figure 3 and Table 3. All samples have significant differences. The NSC has the highest thermal peak at 183.87°C. In the second place, alcalase-soluble collagen (LSC) had one endothermal peak at 153.74°C. The third place is ASC, which had two endothermal peaks at 147.92°C and 169.57°C. The last place is the BSC sample, which had one endothermal peak at 146.14°C.

**Table 2.** Peak positions of FTIR spectra of garut sheepskin collagen.

Band	Wavenumbers (cm-1)			
	BSC	LSC	NSC	ASC
Amida A	3,327	3,401	3,320	3,458
Amida B	2,958	2,930	2,924	2,960
Amida I	1,661	1,656	1,658	1,643
Amida II	1,535	1,535	1,550	1,546
Amida III	1,240	1,245	1,240	1,245

**Table 3.** Thermal stability of collagen isolated using various enzymatic methods.

Extracting agent	Denaturation temperature	Melting temperature
CH_3_COOH + Neutrase (NSC)	-	183.87°C
CH_3_COOH+ Alcalase (LSC)	-	153.74°C
CH_3_COOH+ Bromelain (BSC)	-	146.14°C
CH_3_COOH (ASC)	147.92°C	169.57°C
Commercial type I collagen [21]	200°C	225°C

### Yield of extracted collagen

The yield of extracted Garut sheepskin collagen is shown in Table 4. Bromelain-soluble collagen (BSC) and alcalase-soluble collagen (LSC) have the highest total yield compared to the collagen extracted with other solvents, which reached 37.07 ± 0.61% and 33.66 ± 1.32, respectively. On the other hand, NSC has the lowest total yield and reached 15.69 ± 0.47%. There are no significant differences between NSC and ASC yield percentages. It means that the neutrase enzyme is inefficient in extracting the sheepskin collagen.

### Viscosity of collagen

The viscosity of the Garut sheepskin collagen for all treatments is presented in Table 4. The viscosities of NSC, LSC, BSC, and ASC were 3.90 ± 0.00, 3.45 ± 0.03, 3.12 ± 0.00, and 3.42 ± 0.00 cP, respectively. Bromelin-soluble collagen has the lowest, and neutrase-soluble collagen has the highest viscosity value. Alcalase-soluble-collagen and ASC viscosity values seem to have a slight difference but are still significant for the analysis (*p<0.05*).

### pH value

The pH value of the Garut sheepskin collagen for all treatments was explained in Table 4. The pH of NSC, LSC, BSC, and ASC were 4.76 ± 0.01, 4.87 ± 0.03, 4.01 ± 0.01, and 4.42 ± 0.02, respectively. The analysis results show significant differences in every sample (*p < *0.05).

## Discussion 

Collagen is one of the most needed ingredients in manufacturing food products, medicine, or cosmetics. Garut sheepskin can be an alternative ingredient for producing halal collagen. The quality of local livestock collagen should also be considered by knowing the characteristics of the collagen and comparing it with the standard characteristics of collagen that have been widely traded in the market. Exploration of different analyses such as molecular weight, thermal stability, functional groups of collagen, total yield, viscosity, and pH are needed to determine the quality of collagen, so these analyses were conducted in this study.

Overall, the molecular weight motifs of collagen extracted with various types of solvents, either only with acid or a combination of acid and enzymes, were not significantly different (Fig. 1). Reátegui-Pinedo et al. [17] stated that the molecular weight of the α1 chain of commercial collagen type I was 120 kDa, and the molecular weight of the α2 chain was 116 kDa. Other references Abraham et al. [18] stated that commercial collagen type I of the Sigma brand has a molecular weight of α1 of 200 kDa and α2 of 116 kDa. The results obtained from this study are still in the molecular weight range of type I collagen from previous journal literature.

The pattern of functional groups in Garut sheepskin collagen for all treatments looks similar. It has five Amide bands, defined as collagen structure, comprising amides A, B, I, II, and III (Fig. 2 and Table 2). Gao et al. [19] stated that amide A is formed due to N-H stretching vibrations, while amide B is formed from the asymmetrical stretching of CH2. Amide I shows stretching vibrations of carbonyl groups (C=O bonds) and is related to the secondary structure of proteins. Amide I comprises four protein secondary structure components: α-helix, β-sheet, β-turn, and irregular structure. Amide II shows N-H bending and C-H stretching, while amide III shows intermolecular interactions in collagen related to C-N stretching and N-H bending. The functional group of commercial collagen is reported in Reátegui-Pinedo et al. [17], which stated that commercial collagen has Amide A at wavenumber 3,304, Amide B at wavenumber 2,925, Amide I at wavenumber 1631, Amide II at wavenumber 1,547 cm^−1^ and Amide III at 1235 cm^−1^. The FTIR analysis results for all samples in this study are still in the range amount according to the literature.

Functional group absorbance of Garut sheepskin collagen with acid and enzymatic extraction method has been determined (Fig. 3). According to Kozlowska et al. [20], the first peak is associated with water loss, which causes shrinkage of the collagen fibers and reduces their length to one-third of their original length, called the shrinkage temperature (Ts). Faralizadeh et al. [21] stated that this second peak is relevant to changes in the cross-linked structure of collagen, which leads to the denaturation of the collagen triple helical structure and is called the melting temperature (Tm). It can be concluded from Figure 3. that the thermal stability of collagen from Garut sheepskin is lower than commercial collagen, which was stated by Sarti and Scandola [22] that the melting point (Tm) of Type I commercial collagen is 225°C and the denaturation temperature was around 200°C. On the other hand, Ferraro et al. [23] stated that collagen from several parts of bovine bone has a melting peak starting from 162.42 to 180.01°C.

The higher the melting temperature, the better the quality of collagen because it is resistant to heat. Gauza-Włodarczyk et al. [24] stated that the difference between the studied materials’ denaturation temperatures may result from the hydroxyproline content. The content of this amino acid is related to the collagen’s thermal stability. The hydroxyproline is a specific amino acid of collagen that has a thermal stabilizing effect achieved by forming –O–H…O=C= hydrogen bonds. Furthermore, the stabilizing effect of hydroxyproline is also due to specific stereochemical characteristics of the pyrrolidone ring.

The BSC has the highest total collagen yield. On the other hand, the neutrase enzyme was ineffective at extracting collagen from sheepskin. The NSC has a lower yield of collagen than ASC. However, the differences in both total yields are insignificant (Table 4). Wu et al. [25] stated that the enzymatic method can increase collagen yield rather than just using acid for extraction. However, in the acid-pepsin extraction method, the swelling mechanism of the sample in an acidic solution makes pepsin cleave the cross-linked molecules efficiently at the telopeptide region without affecting the integrity of the triple helical structure, all those processes resulting in a more significant yield compared to the acid extraction method. The difference in yields is also due to many inter-chain and intra-chain crosslinks at the telopeptide region of the collagen. Despite pepsin for isolating collagen, the reference of several enzymes is still limited. However, Devita et al. [10] found that 0.5 M CH_3_COOH containing bromelain produced a high collagen yield, similar to pepsin-soluble collagen.

**Table 4.** Collagen yield, viscosity, and pH were isolated from Indonesia local sheepskin via various enzymatic methods.

Extracting agent	Total yield (%)	Viscosity (cP)	pH
CH_3_COOH + Neutrase (NSC)	15.69 ± 0.47^a^	3.90 ± 0.00^d^	4.76 ± 0.01^c^
CH_3_COOH+ Alcalase (LSC)	33.66 ± 1.32^b^	3.45 ± 0.03^c^	4.87 ± 0.03^d^
CH_3_COOH+ Bromelain (BSC)	37.07 ± 0.61^c^	3.12 ± 0.00^a^	4.01 ± 0.01^a^
CH_3_COOH (ASC)	17.03 ± 0.36^a^	3.42 ± 0.00^b^	4.42 ± 0.02^b^
Commercial type I collagen [21]	-	-	6.52 ± 0.04

^a,b,c,d^Different superscript in the same column indicated significant differences.

The viscosity result in this study (Table 4) was similar to Shon et al. [26], who reported that skatefish skin (*Raja kenojei*) collagen extract has viscosity values ranging from 1.8 to 3.4 Cp. The BSC has the lowest viscosity value. On the other hand, NSC has the highest viscosity value. Ahmad et al. [27] stated that a difference in the molecular weight and molecular size distribution of the protein molecules might contribute to the high viscosity. Proteins with high molecular weight have higher viscosity values. Hadfi and Sarbon [28] state that several factors affect viscosity, such as solution temperature and hydrogen bonds.

The result of the pH value in this study was lower than the commercial collagen (Table 4). The decrease in pH value can occur due to the presence of acidic amino acids that appear after the hydrolysis process using enzymes. Furthermore, Devita et al. [10] stated that the pH value of BSC from big-eye tuna skin ranges from 4.30 to 4.40, which is still similar to the pH values in this study. However, Hadfi and Sarbon [18] stated that commercial collagen has a pH of 6.25 ± 0.04. The difference in pH value can be caused by the nature of the amino acids that make up the collagen. Leon-Lopez et al. [29] also stated that the amino acid sequence and distribution of amino acid residues change due to the type and time of hydrolysis. Furthermore, the time of hydrolysis can affect the pH value of collagen.

Based on the results and discussion, we can conclude that Garut Sheepskin collagen was classified as collagen type I. It can be an alternative product for good-quality collagen from local livestock. BSC also showed the most intriguing results compared to the other treatments.

## Conclusion

Several proteases, including neutrase, alcalase, bromelain solvent, and acid solvent (CH_3_COOH), were successfully applied to collagen extraction from the skin of Garut sheep. It was confirmed as type I collagen using SDS-PAGE analysis. Bromelain treatment resulted in the highest collagen yields. FTIR, DSC, pH, and viscosity results are still in the range of type I collagen isolated in several previous studies. The important point of this study is that collagens from Garut sheepskin can be used as an alternative good-quality and halal collagen product. Collagen extracted using a combination of CH_3_COOH and bromelain enzyme showed the most desirable results in almost every characteristic compared to the other treatment.

## List of Abbreviations

FTIR, Fourier transform infra-red; DSC, Differential scanning calorimetry; ASC, Acid soluble collagen; BSC, Bromelain-soluble-collagen; NSC, Neutrase-soluble-collagen; LSC, Alcalase-soluble-collagen; SDS-PAGE, Sodium dodecyl-sulfate polyacrylamide gel electrophoresis; SPSS, Statistical product and service solution; U/gm, Unit per gram; gm, gram.
